# Book Review

**Published:** 1929

**Authors:** 


### Reviews of Books

**Landmarks and Surface Markings of the Human Body.** By L. B. Rawling, F.R.C.S. Seventh Edition. Pp. 97. Illustrated. London: H. K. Lewis & Co. Ltd. 1929. Price 7s. 6d.—This text-book has been so long and favourably known to students and others as a standard and essentially readable guide to the subject with which it deals that it is not surprising to find that few alterations have been made since the appearance of the last edition five years ago: the modifications consist merely in the redrawing of certain of the illustrations dealing with the anatomy of the foot. We have no doubt that the new edition will continue to enjoy the support of those for whom it is written, no less in the future than during the last quarter of a century.

**Endocrine Disorders.** By Hans Curschmann. Pp. viii., 188. Illustrated. London: Oxford University Press. 1929. Price 12s. 6d.—Endocrinology, a very complex subject, the author has treated in a very thorough and painstaking manner. The English version is rather difficult reading, wordy and heavy in style. In his introductory remarks the author lays great stress on the inter-relationships of the various components of the endocrine system, and their possible control by a hypothalamic centre. Judging by the variety of the various glandular proprietary preparations quoted and recommended, some faith is placed on the therapeutic value of extracts other than the thyroid alone, although admittedly there is not much enthusiasm. A very discursive description of the various types of hypothyroidism makes the subdivisions difficult to conceive in their complexity. Diabetes insipidus is dealt with fully and well, and the various factors in endocrine dwarfism are discussed at some length. There is an illuminating section devoted to homo-sexuality, but there is no faith expressed for operative measures in these conditions, nor is any brief held for success in surgical attempts at rejuvenation. Great care and toil has obviously been expended on this work. The book is well turned out, with some excellent photographs, and should repay a careful perusal by anyone interested in this modern and difficult subject.

**A Text-book of the Practice of Medicine.** By Frederick W. Price, M.D., F.R.S. Third Edition. Pp. xxxviii., 1,871. Illustrated. London: Oxford University Press. Price 36s.—Within four years this text-book has secured for itself a leading place among those written in the English language. The third edition, which now appears, includes articles on many subjects which are noticed in the book for the first time. Most of these are commendably brief, but the result is that regrettable growth in bulk which is the inevitable fate of the successful text-book. This in itself is a drawback to the book, and it has also perhaps been responsible for the imperfect printing which makes this edition less delightful to look at than its predecessors were. However, the matter of the book is beyond praise, and it will doubtless continue for many years to hold the high place that it has justly earned among students and practitioners of medicine.

**The Treatment of Rheumatoid Arthritis.** By A. H. Douthwaite, M.D., F.R.C.P. Pp. 80. London: H. K. Lewis & Co. Ltd. 1929. Price 6s.—This monograph should be read by everyone who has to deal with cases of arthritis. The author stresses the importance of early recognition of the disease, and his description of the prodromal symptoms is concise and clear. While recognizing the present unsatisfactory classification of the various forms of chronic arthritis, the writer has done much to clarify the position of the rheumatoid type. He does not support the focal sepsis theory, but considers the disease to be of a constitutional nature (autonomic, endocrine, metabolic), and his methods of treatment are based on those lines. A very interesting book, and one which should lead to further research.

**The Clinical Examination of the Nervous System.** By G. H. Monrad-Krohn, M.D., F.R.C.P. Fourth Edition. Pp. xvi., 209. Illustrated. London: H. K. Lewis & Co. Ltd. 1928. Price 7s. 6d.—Former editions of this book have been reviewed in this *Journal*. That it has now reached a fourth edition shows that it has undoubtedly been useful. Some minor additions and alterations have been made, so that the subject-matter has been brought thoroughly up-to-date, and a short chapter has been added on interpretation of X-ray pictures of the skull. The book can be very strongly recommended as a reliable guide to the examination of the nervous system, both to medical students and practitioners. There are numerous illustrations, both photographic and diagrammatic, which are well chosen.

**Naval Hygiene.** By Thomas Brown Shaw, M.B., Ch.B. Pp. xvi., 365. 24 illustrations. London : Oxford University Press. 1929. Price 21s.—A useful and up-to-date treatise on hygiene as it particularly concerns those responsible for the health of the seaman, both in the Royal Navy and Mercantile Marine. A clearly-printed volume, well illustrated, in which the writer, while disclaiming any attempt to compete with books on general hygiene, deals with the nautical requirements with a thoroughness and clarity which make easy reading. Each section gives a concise account of the difficulties our predecessors had to contend with and the advances that have been made in dealing with them. Chapter xiii., on vermin and insects, is of particular interest, and one can only wish that space allowed of further particulars of some of the rarer diseases thus conveyed. The choice of modern methods of disinfecting ship, clothing and person offered the ship's surgeon is most generous, and to all such the book should prove of great value.

**Indigestion.** By H. J. Paterson, M.D., F.R.C.S. Pp. viii., 153. London : Wm. Heinemann Ltd. 1929. Price 7s. 6d.—It may seem strange that a surgeon should write a book on so medical a subject as indigestion, a book, moreover, which deals in some detail with conditions in which there could never be any question of operation, which gives full particulars of dietetic and medicinal treatment, and makes no attempt to describe surgical procedures. Attention may be called to the description of acute attacks of hypersecretion, usually seen in young adults who have probably some latent organic lesion, and characterized by severe pain and vomiting, maybe collapse and headache, lasting a few hours or days ; to be distinguished from acute toxic gastritis by the plentiful HCl in the vomit even when no food is being taken. Chronic hypersecretion is the condition that gives rise to "hunger pain," and 20 cc. or more of gastric juice will be found even in the fasting stomach. It is just as likely to be due to a diseased appendix as to duodenal ulcer. Mr. Paterson attaches little importance to the administration of alkalies in the treatment of gastro-duodenal ulcer, preferring six weeks in bed on a rigid diet, only milk and eggs being allowed at first. He refuses to believe, and rightly, that big gastric ulcers are capable of healing in a few weeks, in spite of alleged X-ray evidence. In the diagnosis of cancer of the stomach, the author lays stress on the danger of missing an early case, because the patient may show some temporary improvement on rest and dieting. Altogether, a most useful and practical book for everybody's everyday use.

**Westminster Hospital Reports. Vol. 20.** Edited by STANFORD CADE and DONALD PATERSON. Pp. viii., 343. Illustrated. London : H. K. Lewis & Co. Ltd. 1929. Price 7s. 6d.—The principal points of interest are as follows. There is a follow-up of all the cases of cancer over some four years treated by radium, 190 in number. It is pleasant to notice that some treated in 1926 are still alive and well. Excellent photographs are given of an advanced case of mycosis fungoides. Experiments on the cat's heart to study the effect of adrenalin under anæsthesia show that it had a double action, and may cause quickening or fibrillation. It is not surprising therefore that it does not always resuscitate a case of syncope under anæsthesia. The value of X-ray treatment for lymphadenoma is emphasized. There is a discussion on cataract. Twenty-four cases treated by peri-arterial sympathectomy are described. It was found beneficial for Raynaud's disease and for acrocyanosis, and to some extent for chronic ulcer, but not for endarteritis or for causalgia.

**Westminster Hospital Reports. Vol. 21. Radium Practice.** Edited by E. ROCK CARLING. Pp. vii., 258. Illustrated. London : H. K. Lewis & Co. Ltd. 1929. Price 7s. 6d.—Naturally books on the radium treatment of cancer are pouring out of the press just now, and new practical points emerge every few months. This volume contains the lectures delivered in July, 1929, by eighteen members of the staff, and gives more information than any other recent book by British writers. The chapters dealing with technical details of treatment follow much the same lines as those laid down in Mr. Stanford Cade's excellent monograph published earlier in the year, but with certain advances and additions. For instance, for rectal cancer needles screened with 0·8 mm. of platinum are used. The radium bomb has come into action, and to some extent takes the place of surface irradiation or of operation, for instance in cases of glands of the neck. A suprapubic de Pezzer drain is advised when treating an epithelioma of the penis. There is a very interesting chapter on radio-sensitivity, in which it is pointed out that the bony metastases of cancer will often yield excellently to X-rays. Bone sarcoma is radio-resistant, but may be benefited by the bomb. Myeloids may be cured by X-rays. The remarkable statement is made that patients with lymphosarcoma and lymphadenoma are likely to live longer if not treated. Radiation should be reserved for cases with dyspnœa or other distressing symptoms. All growths respond much better to a first dose of radium than to a second. The first ought, therefore, to be adequate.

**The Prevention of Human Tuberculosis of Bovine Origin.** By W. G. SAVAGE, M.D. Pp. viii., 195. London : Macmillan & Co. Ltd. 1929. Price 10s. 6d.—Dr. Savage's able and informative book appears at a most opportune moment, when the question of bovine tuberculosis is receiving the attention of the Government, the veterinary profession and, one may venture to hope, the Farmers' Union. The author, after reviewing the very extensive but very scattered literature on the subject, is left with two impressions : (1) the lack of precise data with which to arrive at a solution, and (2) the piecemeal way in which measures are put into operation without consideration of the unity of the problem. After showing that milk is in the vast majority of cases responsible for the infection of humans by bovine bacilli, Dr. Savage discusses in detail and with reference to all the available statistics the amount of bovine infection, and concludes that 1 per cent. of fatal respiratory and 23 per cent. of fatal non-respiratory human tuberculosis is bovine in origin. The prevalence of tuberculosis in cattle in Great Britain is then discussed, and a rather depressing picture is drawn. No record of routine tuberculin tests is available in this country, abattoir statistics vary greatly in different districts where there are public abattoirs, and in many areas there is no routine inspection of cattle by the local authority. But, as Dr. Savage points out, the crux of the question is the ignorance of the average cow-keeper of the basic facts as to the spread of tuberculosis in dairy stock, and he states that if the active co-operation of agriculturalists cannot be enlisted, then the only solution is efficient pasteurization. It does seem odd, to say the least of it, that the State punishes a butcher who exposes for sale tuberculous meat, but allows a dairyman to sell tuberculous milk without penalty. The book ends with an ideal scheme for the reduction and eventual elimination of bovine tuberculosis which is reasonable, practicable, and not too costly, provided the co-operation of the stock-keeper is enlisted. All depends on that. Dr. Savage is to be congratulated most heartily on this monograph.

**Radium and its Surgical Application.** By H. S. SOUTTAR, F.R.C.S. Pp. 60. Illustrated. London : Wm. Heinemann Ltd. 1929. Price 7s. 6d.—This small book, which can be read through in an hour or two, follows much the same lines as the distinguished author did when he gave a memorable address earlier in the year to the Bristol Medico-Chirurgical Society. The earlier chapters are devoted to a very clear and very fascinating account of the physics of the radium atom, and the methods by which radium can be applied clinically, the plaque, the needle, the bomb, and the radon seed. Valuable directions are given as to the methods and dosage for treating carcinoma of the various organs of the body. The writer has a preference for seeds in some situations where other radium experts prefer needles. We are glad to observe that he now uses spongy rubber sheeting instead of that very uncomfortable substance, Columbia paste, for external radiation. He points out that a growth which is to be treated with radium must not be given X-rays, or it will not respond. It is disquieting to read that extraction of a tooth years after a tongue has been treated by radium for epithelioma may be followed by necrosis of the jaw. Of course, the whole subject is so new, and experience growing so fast, that there is room for much difference of opinion, and a book may be out of date almost as soon as it is published. We think the author is likely to rewrite some of his remarks, and to add others on the subject of screenage. The table on page 15 would give the impression that a 0·5 mm. screen of platinum makes all safe, but expert radium workers are screening more and more, and for the bowel probably 0·8 or 1·0 is desirable. No mention is made of a lead plate to protect the palate in mouth cases.

**Difficult Labour.** By G. E. HERMAN. Seventh Edition. Edited by C. OLDFIELD, M.D., F.R.C.S., F.R.C.P. Pp. xiv., 560. Illustrated. London: Cassell & Company Ltd. 1929. Price 16s.—Dr. Carlton Oldfield is to be congratulated on the present edition of this book, which has always ranked as a classic among midwifery text-books. The mechanical problems of labour are dealt with fully, clearly and skilfully, and some interesting X-ray plates are illustrated. The chapters on hemorrhage are good; Willett's forceps are mentioned in the treatment of placenta prævia, and extreme necessity of immediate treatment in this condition is stressed; a very valuable point this, as there is a growing tendency to send the still bleeding woman into hospital. The chapter on eclampsia has been rewritten and is now up to date, and there is a very short but pithy chapter on the prevention of puerperal sepsis, in which the value of rectal examination is upheld. It is rather surprising that lower segment cæsarean section should be dismissed with a curt mention; but this is a small matter, and one can only praise the reviser and publishers for compressing so much valuable information, both for students and practitioners, in such a small and compact volume.

**The Nervous Child.** By H. C. CAMERON, M.D., F.R.C.P. Fourth Edition. Pp. 249. Illustrated. London: Oxford University Press. 1929. Price 7s. 6d.—The appearance of another edition of this eminently readable and common-sense book must always be welcome. The criticism that while the description is all that is admirable the explanation of symptoms is not so adequately dealt with may still obtain, but since this book has become so widely read by the lay public and the explanation of nervous symptoms in children is still in dispute this is probably a good fault. A new chapter has been added on the underlying disturbances of metabolism in the nervous child. In this the association of nervous prostration and general muscular atonia with ketosis has been described. The origin of ketosis in the exhaustion of carbohydrate reserves and the irregular mobilization of fats with diminution of the P.M. value of the blood is explained, and the logical treatment by restriction of fats and administration of glucose and alkalies is detailed. This is a book which all those concerned in the upbringing of children should have on their shelves.

**The Radium Treatment of Cancer of the Uterus.** By THE CANCER RESEARCH COMMITTEE OF THE MARIE CURIE CLINIC. Pp. 37. Illustrated. London: H. K. Lewis & Co. Ltd. 1929. Price 2s. 6d.—This report deals with the three-and-a-half years' work in this Clinic since the publication of the first report in 1926. The report deals chiefly with cancer of the cervix, the preparatory treatment, technique of application and contra-indications and complications of radium treatment. Few cases only of cancer of the uterine body have been treated, but these are fully dealt with. The statistical table is necessarily incomplete, as the Clinic has not been working for five years, and therefore cannot give any definite "cures." The end results are given as "the number living on October, 1928," and therefore cannot be compared with other statistical "cures." The technique employed is a modification of that used at Stockholm. Intrauterine and vaginal applicators are combined; the uterine applicator containing 50 mgm. radium element with 1 min. platinum screen and the vaginal applicators 25 mgm. radium element in each with 1·33 min. platinum screen, three being used at each application. Three applications are given each of 22 hours, at intervals of 7 and 21 days after the first application. A résumé of techniques used in other clinics is given, and there is a chapter on the comparative results. The whole is unbiased and well compiled.

**Hypochondria.** By R. D. GILLESPIE, M.D., M.R.C.P. Pp. 104. London: Kegan Paul, Trench, Trubner & Co. Ltd. 1929. Price 2s. 6d.—This essay on hypochondria is to be commended to every thinking physician. It enjoys all the clarity of expression and philosophical outlook and literary erudition which we have come to expect from Dr. Gillespie. The first chapter deals with the history of what was known in the eighteenth century as the English disease. Dr. Gillespie differentiates hypochondria from anxiety states and hysteria, points out that it occurs almost exclusively in men and is singularly resistant to psychotherapy. He points out that it occurs in egocentric personalities possibly definitely schizoid. Indications of narcissism and unconscious homo-sexuality are often to be discovered. Just as the eighteenth century was an age of hypochondria, so is the present, and the author thinks this may be due in both cases to a breaking with old standards without the establishment of new codes and philosophies of life to which the personality may adjust.

**Cancer.** By G. JEANNENEY. Translated by J. GIBSON, M.D., and J. H. WATSON, F.R.C.S. Pp. xiv., 186. Illustrated. London: H. K. Lewis & Co. Ltd. 1929. Price 7s. 6d.—Cancer presents a problem prominent in the mind of every member of our profession. Many medical men have ample opportunity of gaining practical knowledge of this disease daily, if not hourly, and are thus equipped with special knowledge of the various problems connected with it, especially those of early diagnosis and appropriate treatment. The majority are called upon to conduct efficiently any case of cancer encountered in the course of general practice. It is to these and to senior students that M. Jeanneney's book should appeal. The opening part deals with the general pathology of cancer, including ætiology, and modern treatment. The remaining part gives a brief description of the disease as it occurs in each particular part of the body. The importance of early diagnosis is stressed throughout, and helpful points are given to facilitate this. Where there is reasonable chance of success, the author advises surgical removal of the growth in all cases, combined with radium therapy when necessary. Radium alone, X-rays, lead and other remedies are described in proportion to their promise of future success, some receive only a passing reference. The translation is somewhat literal, but this adds to the fascination of a book whose style only serves to increase one's admiration for an author who has the audacity to present so large a subject in so small a space.

**Artificial Sunlight and its Therapeutic Uses.** By F. H. HUMPHRIS, M.D., F.R.C.P. Fifth Edition. Pp. xxii., 340. Illustrated. London : Oxford University Press. 1929. Price 10s. 6d.—This excellent book has in *five* years passed through five editions, which in itself shows that it had met a demand among those who are interested in gaining a knowledge of treatment by artificial sunlight. The fifth edition is made necessary by the various improvements in apparatus, and embodies descriptive detail of the latest models in lamps and electrodes. It discusses, informingly, both the mercury vapour and carbon tungsten type of lamp. One of its most valuable points is that Dr. Humphris makes no extravagant claim for the therapeutic uses of artificial sunlight. The book is written in non-technical language, is comprehensive in character, and is an excellent guide to those practitioners who wish to add actinotherapy to their armamentarium. It clearly indicates for what diseases it may be advantageously used, and is eminently suitable for the consulting-room of any practitioner who would be conversant with the subject.

**The Treatment of Fractures.** By LORENZ BÖHLER, M.D. Pp. 185. Illustrated. Vienna : Wilhelm Maudrich. 1929. Price 21s.—Dr. Böhler describes the methods which are in vogue at the Accident Insurance Hospital in Vienna. They differ so completely from the usual practice in British institutions that they merit the very careful study of every British surgeon. Some of the chief points of differences are : (1) The abandonment of general anæsthesia in favour of local, and, to a less extent, of regional and spinal anæsthesia ; (2) the use of screw-traction apparatus in the reduction of fractures ; (3) the application of unpadded plasters to maintain reduction and for the ambulatory treatment of fractures of the lower limb. The open operative reduction of fractures is condemned as unnecessary and dangerous, and is reserved for a few special cases, such as the patella and certain fractures in the immediate neighbourhood of joints. While Dr. Böhler is an advocate of early active movement wherever this is possible, he considers that massage and passive movement are not called for, and that they are frequently productive of harm. The one and only essential, according to his teaching, would seem to be accurate reduction of every fracture and the maintenance of the bones in good position until firm union has taken place. All the apparatus required for the treatment of fractures has been standardized, and all parts are made interchangeable. A careful and detailed description is given of every article which

Y

must be put out when any given fracture is to be treated, and the steps taken are very simply and clearly described. The illustrations are excellent, and help the text greatly. The printing is good, but there are a good many typographical errors, and the English is not fluent, the book being very obviously a translation.

**The Treatment of Fractures and Dislocations in General Practice.** By C. M. PAGE, M.S., F.R.C.S., and W. R. BRISTOWE, F.R.C.S. Third Edition. Pp. xviii., 284. Illustrated. London: Oxford University Press. 1929. Price 14s.—The continued call for this clear and concise treatise on fractures is pleasantly proved by the appearance of a third edition. Like its predecessors it is beyond praise. The doctor is often concerned with fractures owing to the frequent accidents of modern motor transport, and he will find no better friend than this book when dealing with such cases. An interesting error of description is repeated in this edition and more noticeably. The line of fracture of the humerus, shown with such emphasis in the figure on page 93, passes through the "surgical neck" and not, as stated in the foot-note, through the "anatomical neck." A new illustration is needed to portray the latter variety of fracture. In former editions the line of fracture was obscure, but now the error is patent. Some modifications of the view of the etiology of Volkmann's ischæmic contracture seems desirable. Possibly, too, the condition demands rather more space. The reader receives the dominating impression that the ischæmia is caused by tight bandaging, and is thus the outcome of treatment, whereas it occurs not rarely in the entire absence of external pressure during post-accident treatment, *i.e.* it is then a complication of the initial injury and not due to treatment. Moreover, even where bandaging has been employed, the trouble may have already been developing independently and not from the doctor's handling. It seems due to truth, as well as to our much-tried profession, that impressions should not be maintained in surgical text-books that are calculated to damn irretrievably the doctor in whose practice Volkmann's ischæmic contracture may declare itself at any moment.

**Movable Kidney.** By W. BILLINGTON, M.S., Ch.M., F.R.C.S. Second Edition. Pp. xiv., 177. Illustrated. London: Cassell & Company Ltd. 1929. Price 12s. 6d.—There are few men better qualified than he to write it, and we welcome a second edition of *Movable Kidney* by Billington. It deserves a first place among surgical monographs. It deals with a subject of mixed repute and successfully raises it, by fair reasoning and a comely breadth of consideration, to a place alongside the standard procedures of surgery. To the description of the actual undertaking for stabilization of the kidney the author allows but a small part of the book. The operation is fully and admirably illustrated. For statistical evidence of his results he adopts the novel plan of presenting the figures for the criticism of a foreign professor, and quotes freely from his report in the last chapter. While he only claims 70 per cent. successes, this represents a substantial boon to mankind, when it is noted that complications and mortality are almost unknown in the author's hands. That surgeons in general do not emulate this author is possibly due to the fact that, whereas many kidneys move unduly, only a few of their owners will benefit by nephropexy. Good results depend, not on the diagnosis of mobility, but on the careful correlation of this to the patient's symptoms and constitution. To distinguish the suitable case calls for consideration of matters more subtle and involved than the simple issues upon which decision to operate is usually based. Hence the stumbling-block among average surgeons, which prevents them securing the successes of this author and advocate. Hence, too, the author's wise allocation of five, out of fourteen, chapters to the symptomatology, not to mention a chapter on visceroptosis. He is to be congratulated on a comprehensive study of the subject, cogent in argument and pleasing in style, which will bring clarity, and in some cases conviction, to the minds of medical men who are mostly hazy about it.

**Œsophageal Obstruction.** By A. L. Abel, M.S., F.R.C.S. Pp. xii., 234. Illustrated. London: Oxford University Press. 1929. Price 30s.—It would seem to be difficult to think of any œsophageal disease of which obstruction is not a prominent feature; this work might well have been titled "Diseases of the Œsophagus." It presents a comprehensive survey of the subject from congenital abnormalities to cancer. One is perhaps inclined to turn first to the latter section, since malignant disease accounts for much more than half of all œsophageal disorders. The author is pessimistic about present methods, and advocates external operation; unfortunately, all his patients so treated have died, the almost invariable sequel of such operations. We cannot agree that cancer of the œsophagus causes early symptoms; it is very rare indeed to see an early case, and this constitutes the principal bar to extensive operation. The book is extremely well got up and profusely illustrated, and should find a place in every reference library.

